# Supplementary material for: Constituents from the Seeds of Sophora Alopecuroides L
Source: Molecules. 2020 Jan 19;25(2):411. doi: 10.3390/molecules25020411 (PMC7024312; doi:10.3390/molecules25020411)
Supplement: Supplementary file 1 [file molecules-25-00411-s001.pdf]

SUPPLEMENTARY MATERIAL

Catalogue

Figure S1. ^{1}H NMR (600 MHz, DMSO-d_6) spectrum of the new compound **1.**....1
Figure S2. ^{13}C NMR (150 MHz, DMSO-d_6) spectrum of the new compound **1.**....1
Figure S3. HSQC spectrum of the new compound **1.**....2
Figure S4. HMBC spectrum of the new compound **1.**....2
Figure S5. ^{1}H-^{1}H COSY spectrum of the new compound **1.**....3
Figure S6. NOESY spectrum of the new compound **1.**....3
Figure S7. Enlarged NOESY spectrum of the new compound **1.**....4
Figure S8. HRESIMS spectrum of the new compound **1.**....4
Figure S9. The chromatogram of the glucose standard and hydrolyzation product of **1**....5
Figure S10. ^{1}H NMR (600 MHz, CD_3OD) spectrum of the new compound **2.**....5
Figure S11. ^{13}C NMR (150 MHz, CD_3OD) spectrum of the new compound **2.**....6
Figure S12. HSQC spectrum of the new compound **2.**....6
Figure S13. HMBC spectrum of the new compound **2.**....7
Figure S14. ^{1}H-^{1}H COSY spectrum of the new compound **2.**....7
Figure S15. NOESY spectrum of the new compound **2.**....8
Figure S16. Enlarged NOESY spectrum of the new compound **2.**....8
Figure S17. HRESIMS spectrum of the new compound **2.**....9
Figure S18. The chromatogram of the glucose standard and hydrolyzation product of **2**....9
Figure S19. ^{1}H NMR (600 MHz, CD_3OD) spectrum of the new compound **3.**....10
Figure S20. ^{13}C NMR (150 MHz, CD_3OD) spectrum of the new compound **3.**....10
Figure S21. HSQC spectrum of the new compound **3.**....11
Figure S22. HMBC spectrum of the new compound **3.**....11
Figure S23. ^{1}H-^{1}H COSY spectrum of the new compound **3.**....12
Figure S24. NOESY spectrum of the new compound **3.**....12
Figure S25. Enlarged NOESY spectrum of the new compound **3.**....13
Figure S26. HRESIMS spectrum of the new compound **3.**....13
Figure S27. The chromatogram of the glucose standard and hydrolyzation product of **3**....14
Figure S28. ^{1}H NMR (600 MHz, CD_3OD) spectrum of the new compound **4.**....14
Figure S29. ^{13}C NMR (150 MHz, CD_3OD) spectrum of the new compound **4.**....15
Figure S30. HSQC spectrum of the new compound **4.**....15
Figure S31. HMBC spectrum of the new compound **4.**....16
Figure S32. NOESY spectrum of the new compound **4.**....16
Figure S33. HRESIMS spectrum of the new compound **4.**....17
Figure S34. ^{1}H NMR (600 MHz, CD_3OD) spectrum of the new compound **5.**....17
Figure S35. ^{13}C NMR (150 MHz, CD_3OD) spectrum of the new compound **5.**....18
Figure S36. HSQC spectrum of the new compound **5.**....18
Figure S37. HMBC spectrum of the new compound **5.**....19
Figure S38. NOESY spectrum of the new compound **5.**....19
Figure S39. HRESIMS spectrum of the new compound **5.**....20

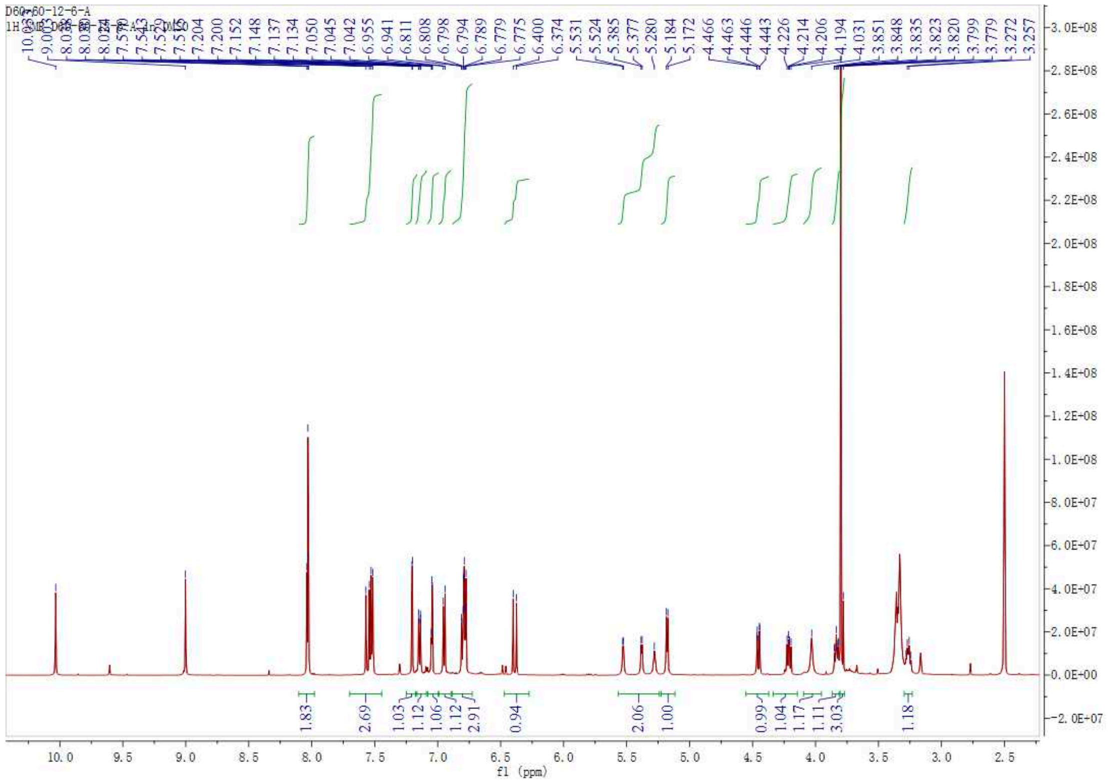

Figure S1. ^{1}H NMR (600 MHz, DMSO-d_6) spectrum of the new compound **1.**

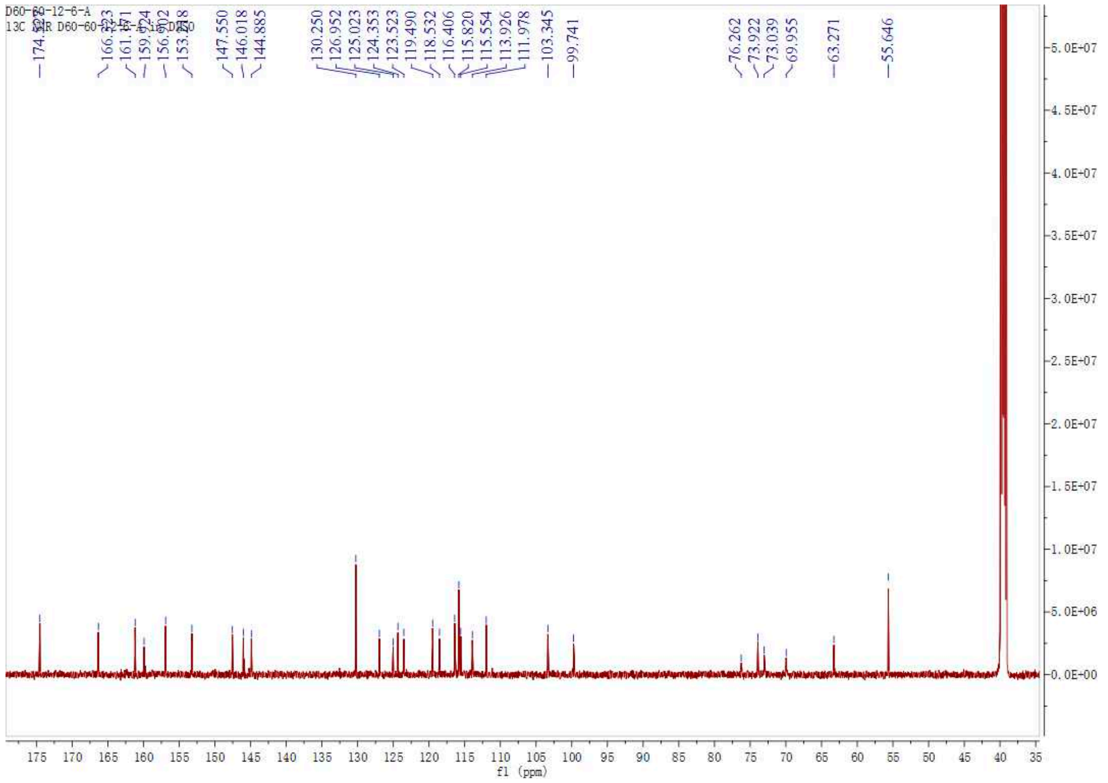

Figure S2. ^{13}C NMR (150 MHz, DMSO-d_6) spectrum of the new compound **1.**

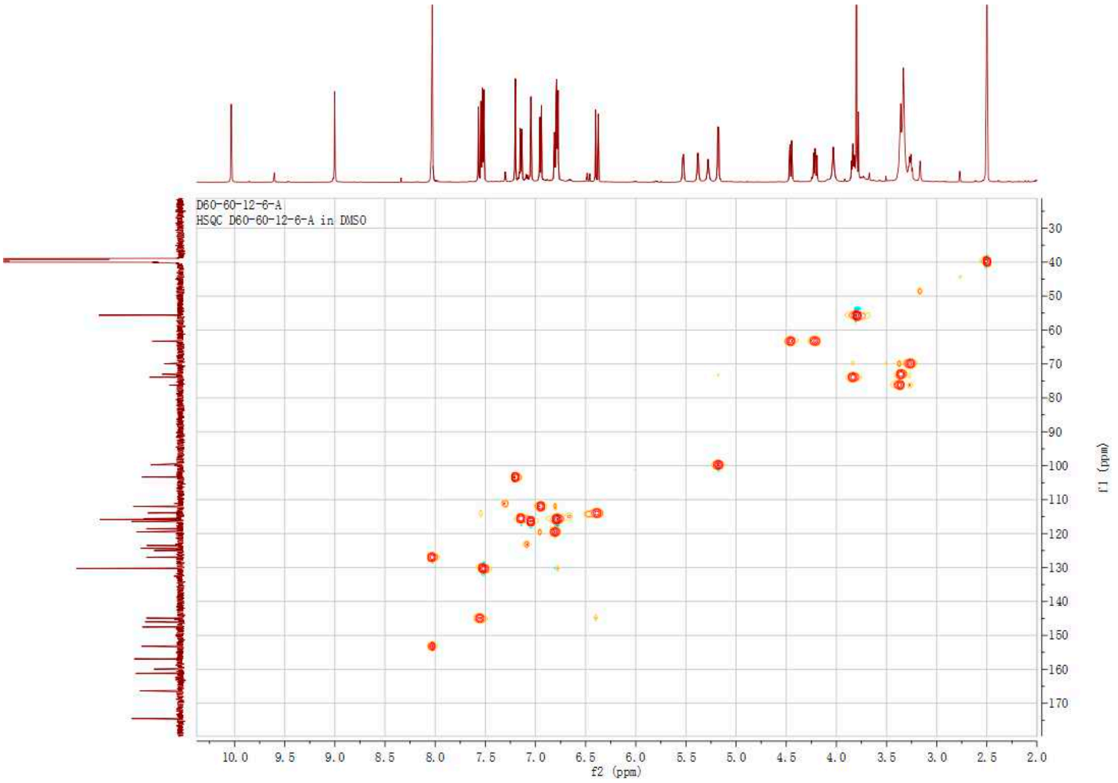

Figure S3. HSQC spectrum of the new compound **1.**

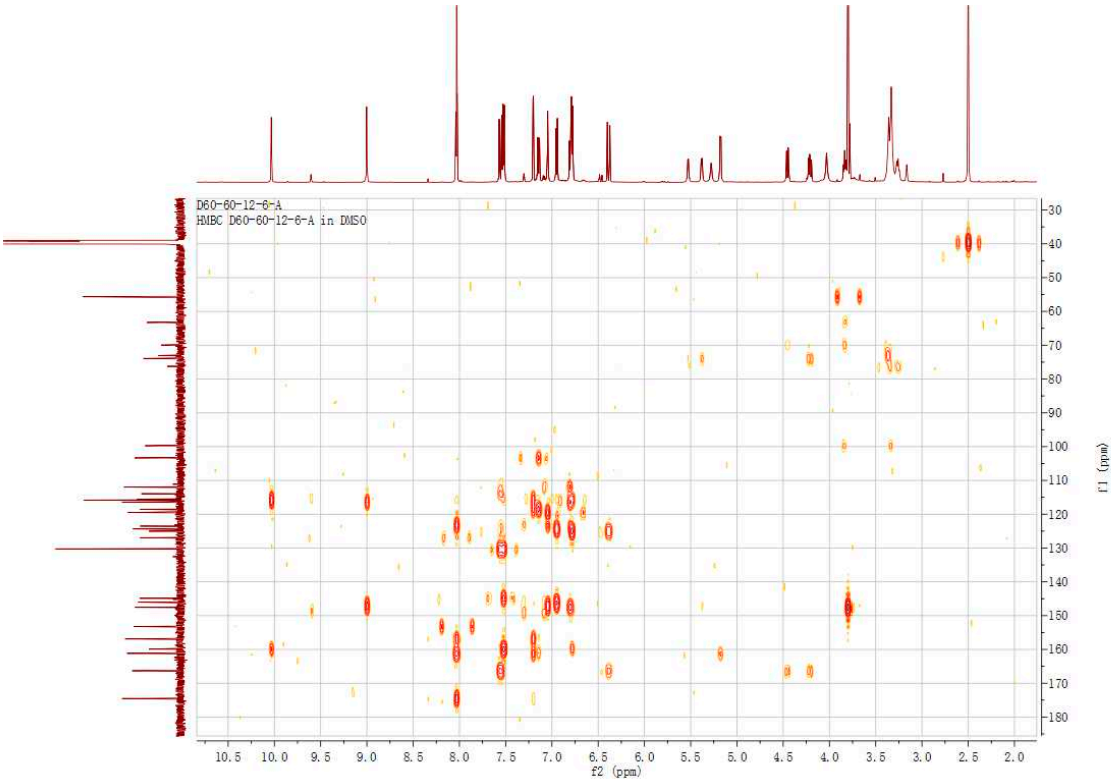

Figure S4. HMBC spectrum of the new compound **1.**

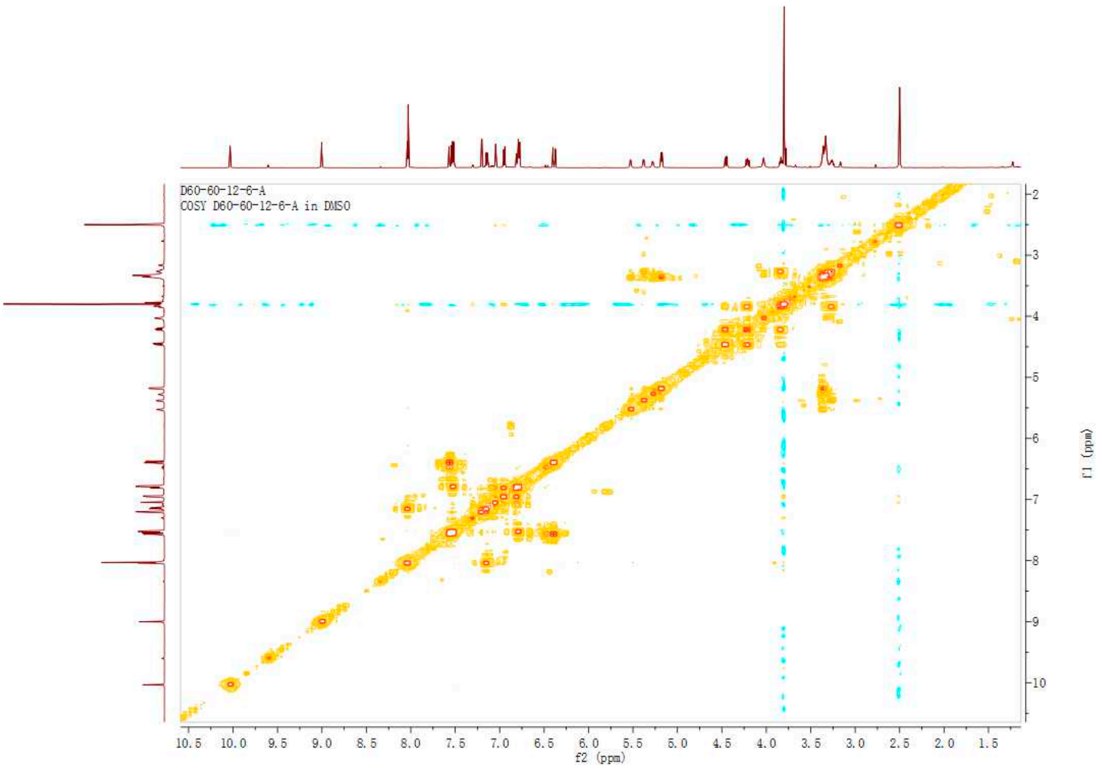

Figure S5. ^{1}H-^{1}H COSY spectrum of the new compound **1.**

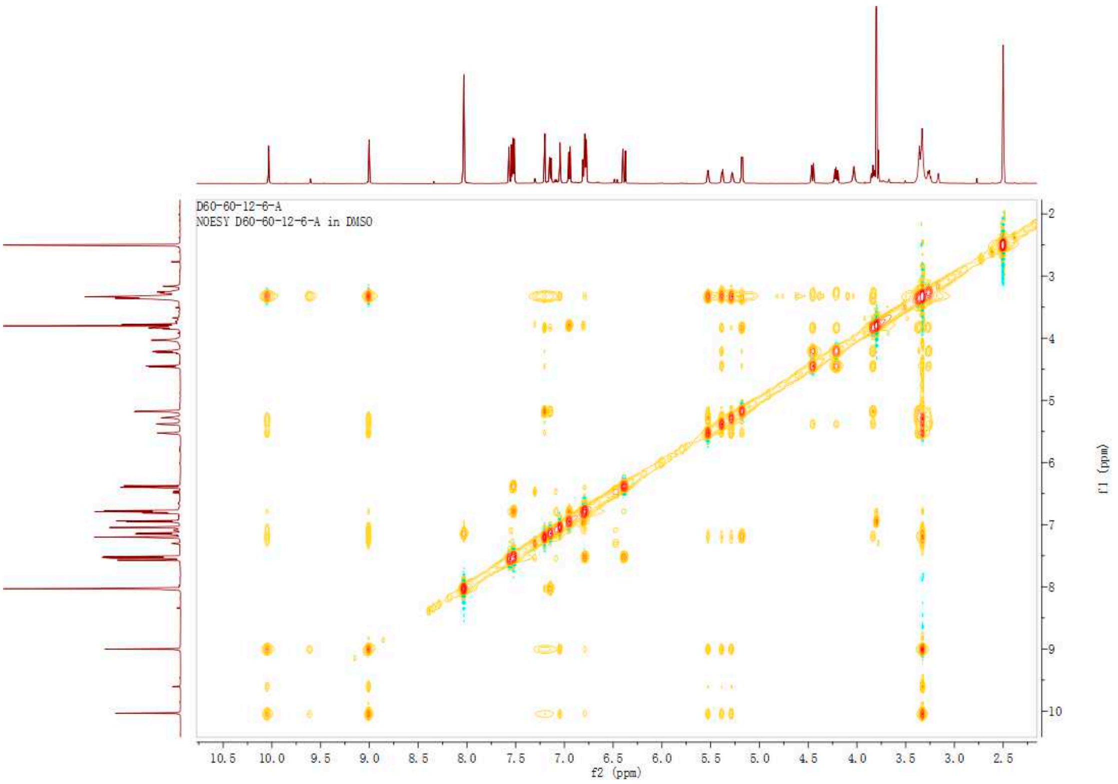

Figure S6. NOESY spectrum of the new compound **1.**

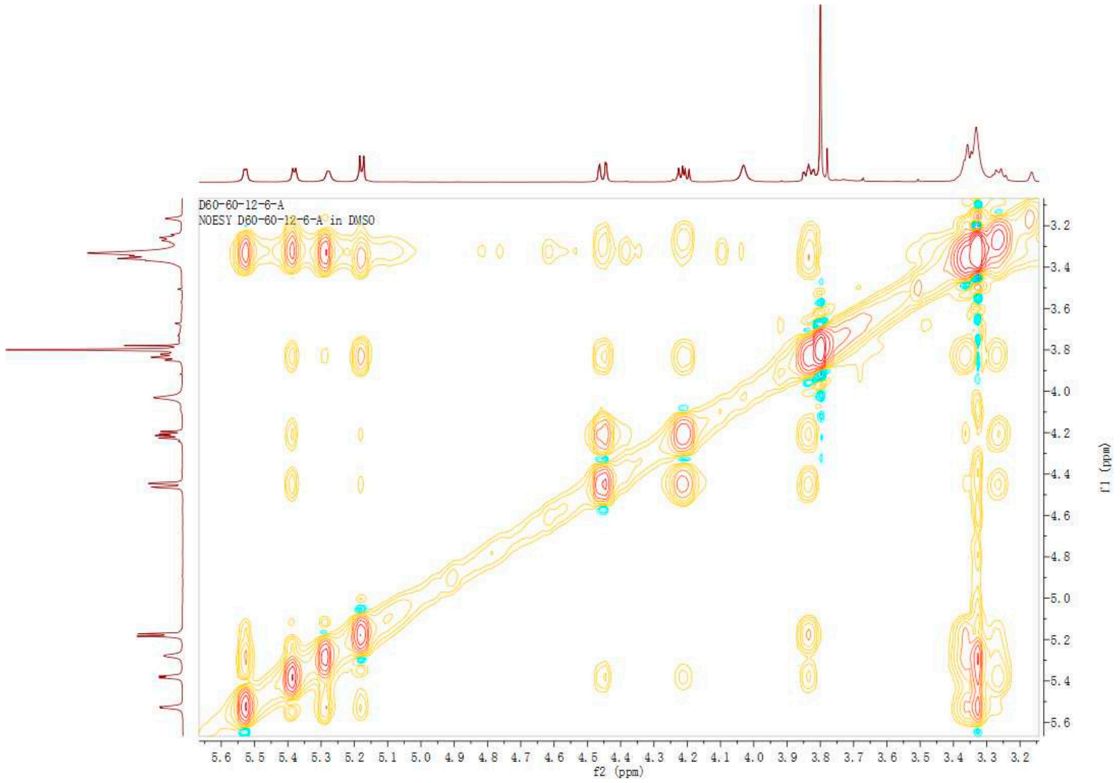

Figure S7. Enlarged NOESY spectrum of the new compound **1.**

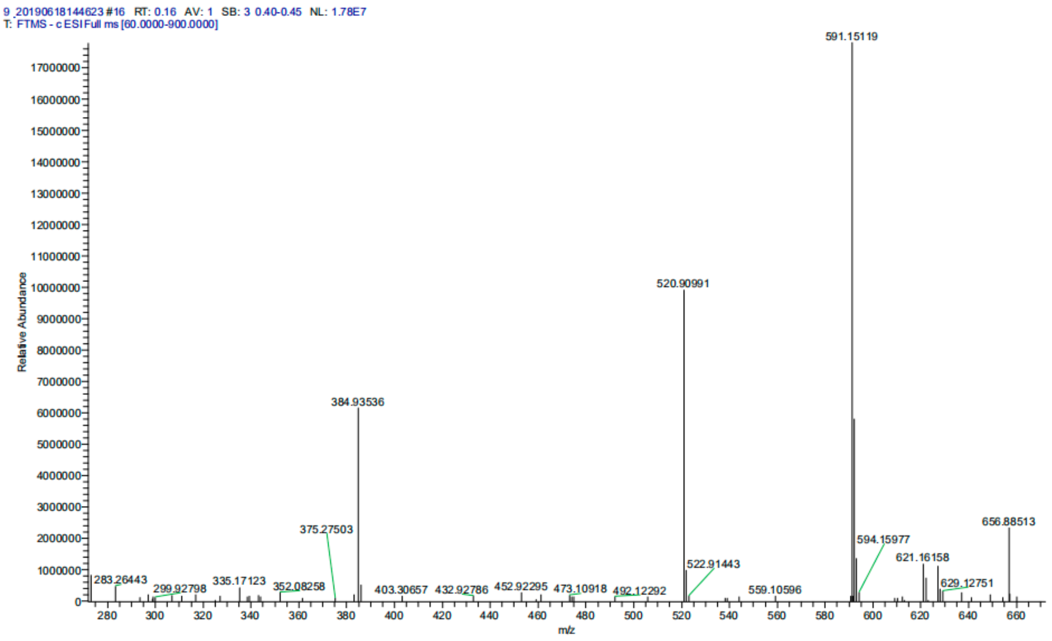

Figure S8. HRESIMS spectrum of the new compound **1.**

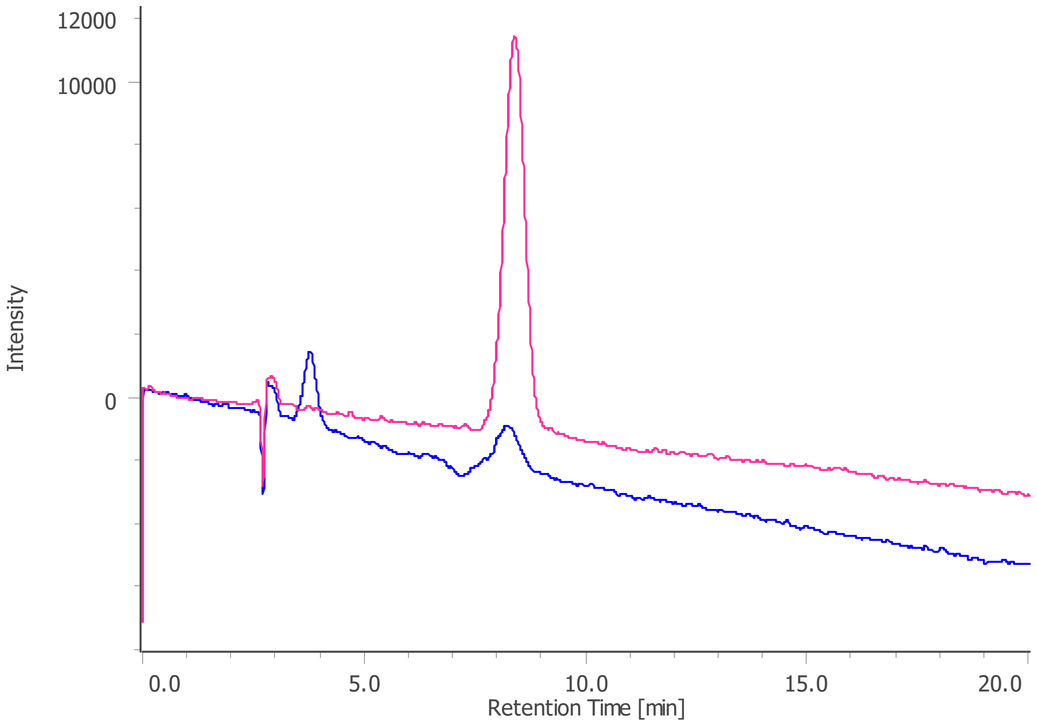

Figure S9. The chromatogram of the glucose standard and hydrolyzation product of **1**

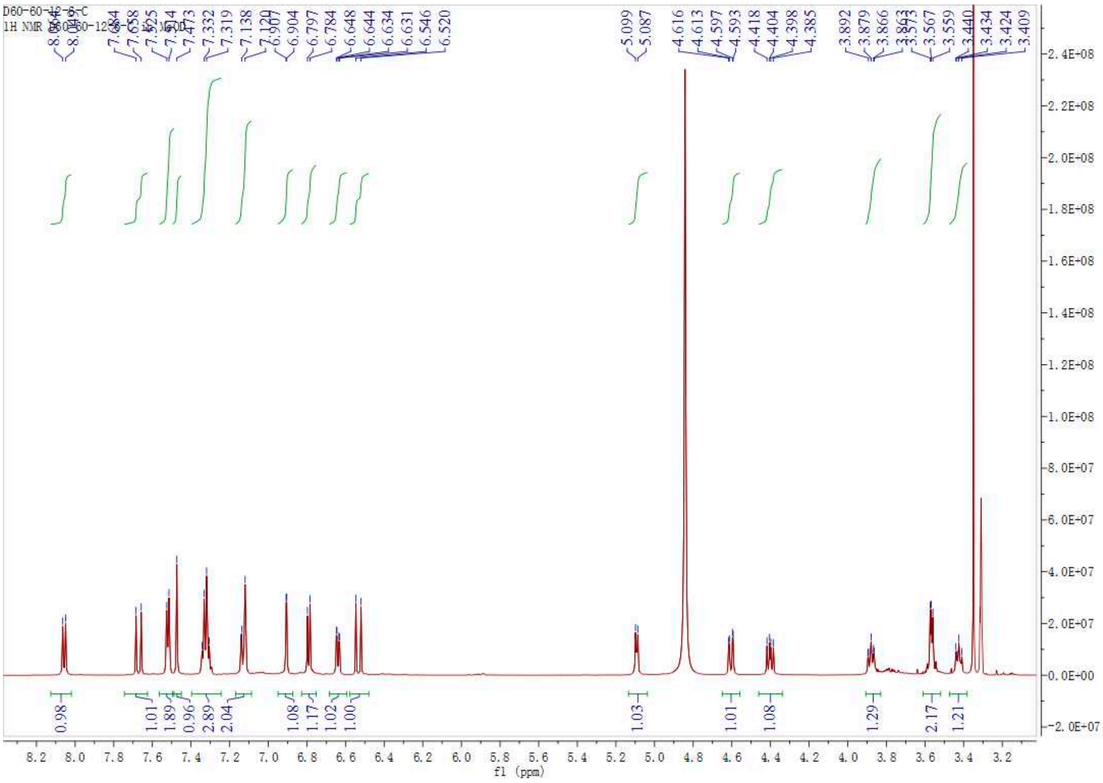

Figure S10. ^{1}H NMR (600 MHz, CD_3OD) spectrum of the new compound **2.**

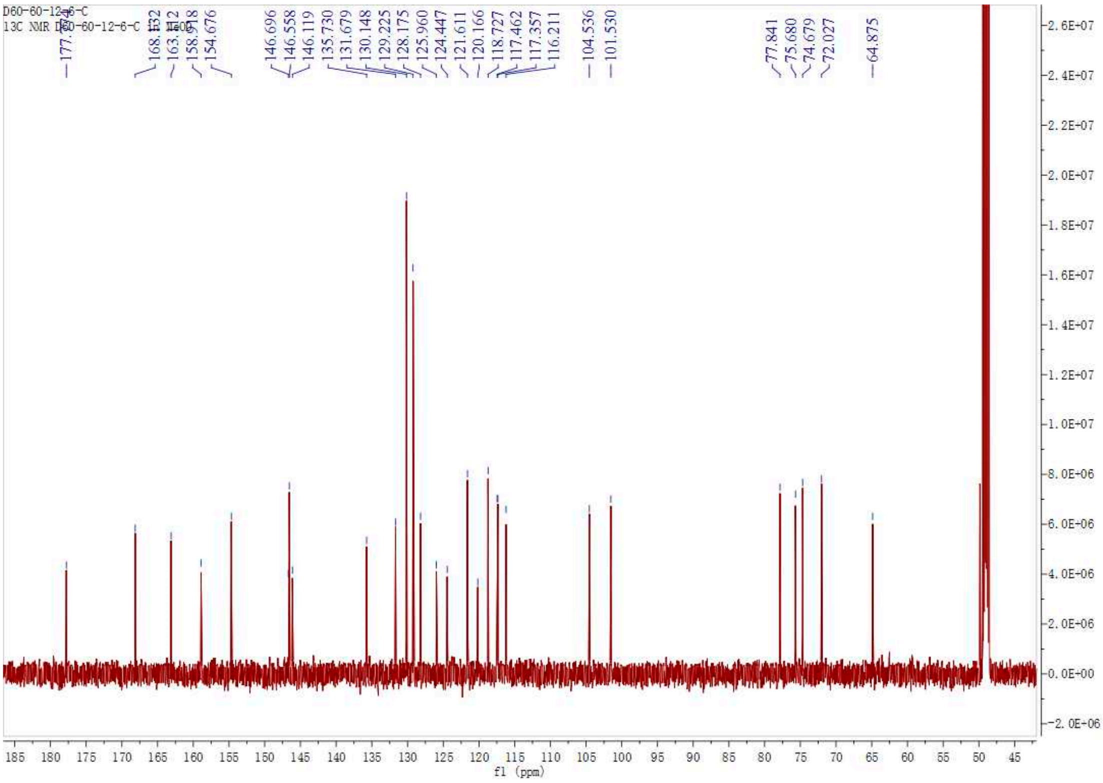

Figure S11. ^{13}C NMR (150 MHz, CD_3OD) spectrum of the new compound **2.**

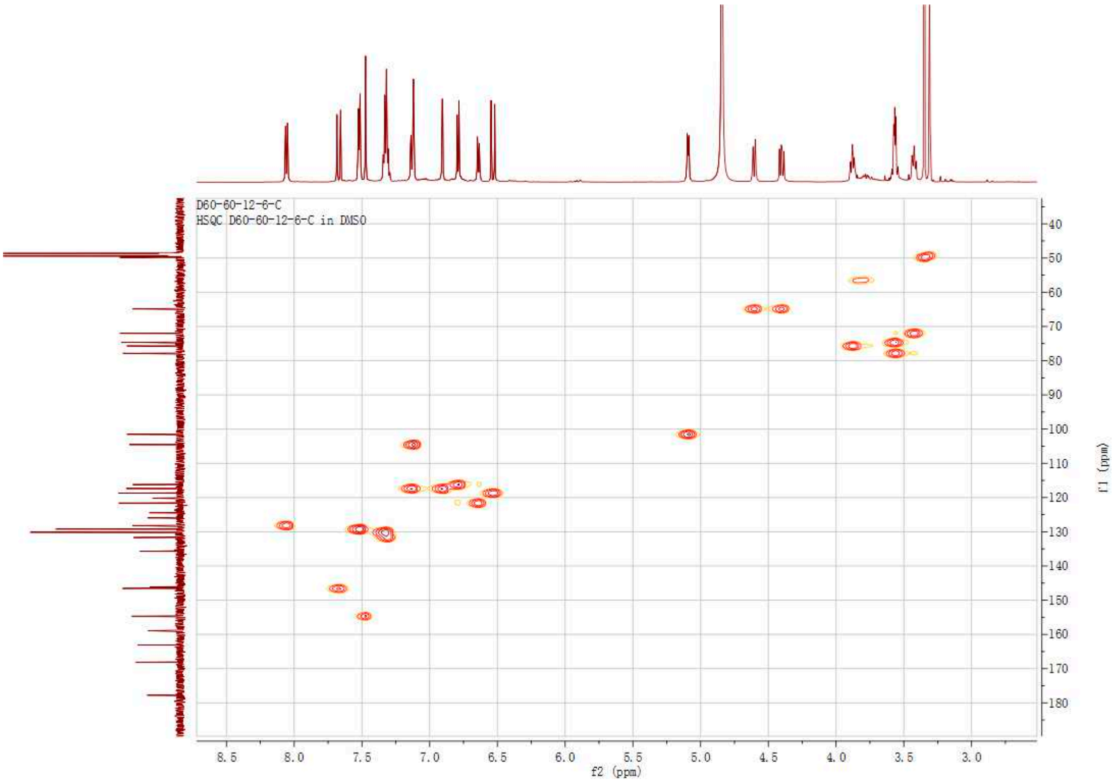

Figure S12. HSQC spectrum of the new compound **2.**

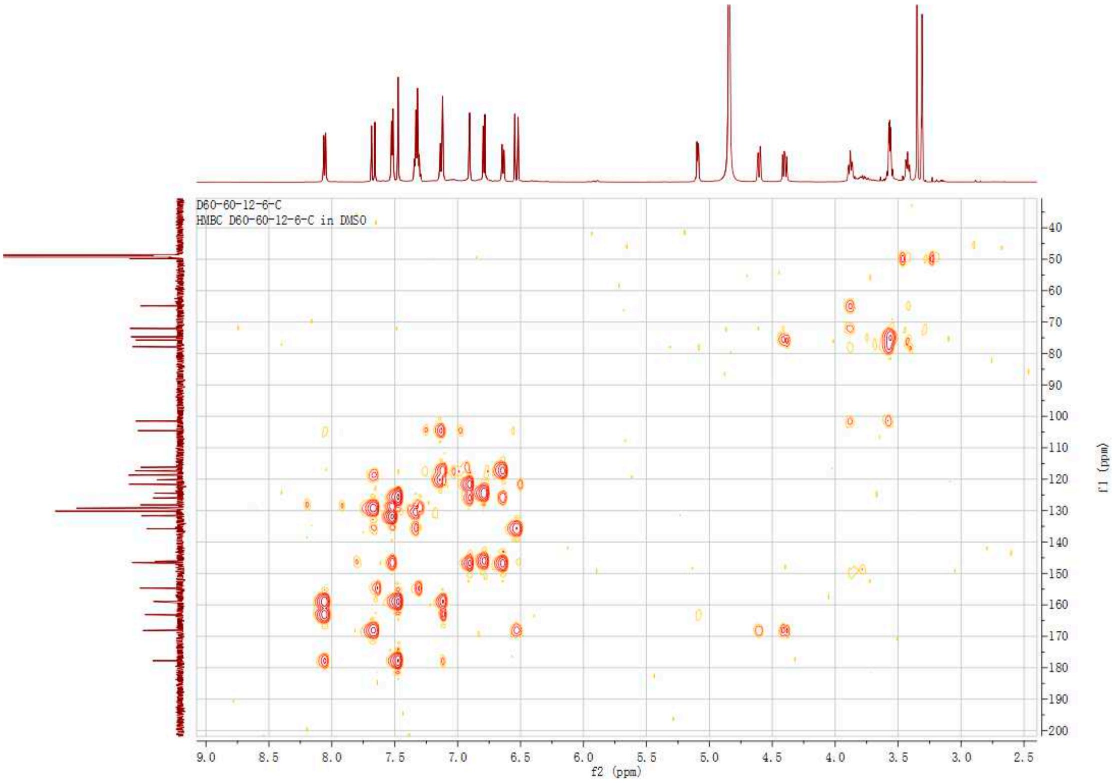

Figure S13. HMBC spectrum of the new compound **2.**

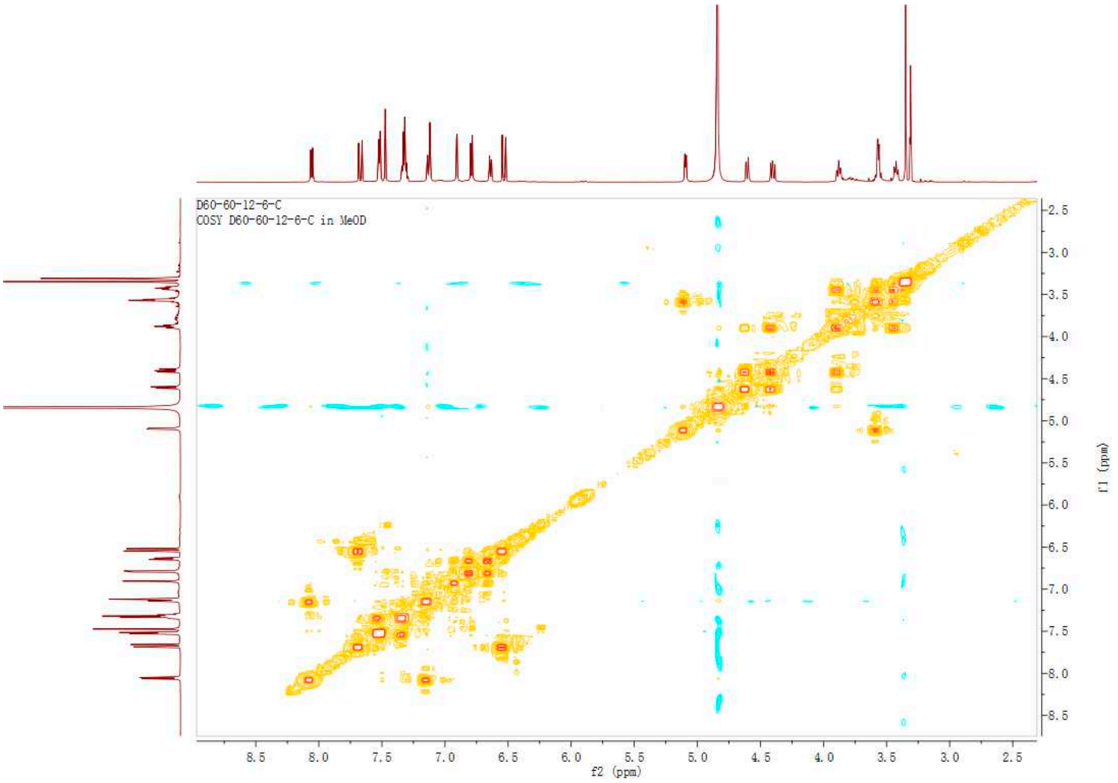

Figure S14. ^{1}H-^{1}H COSY spectrum of the new compound **2.**

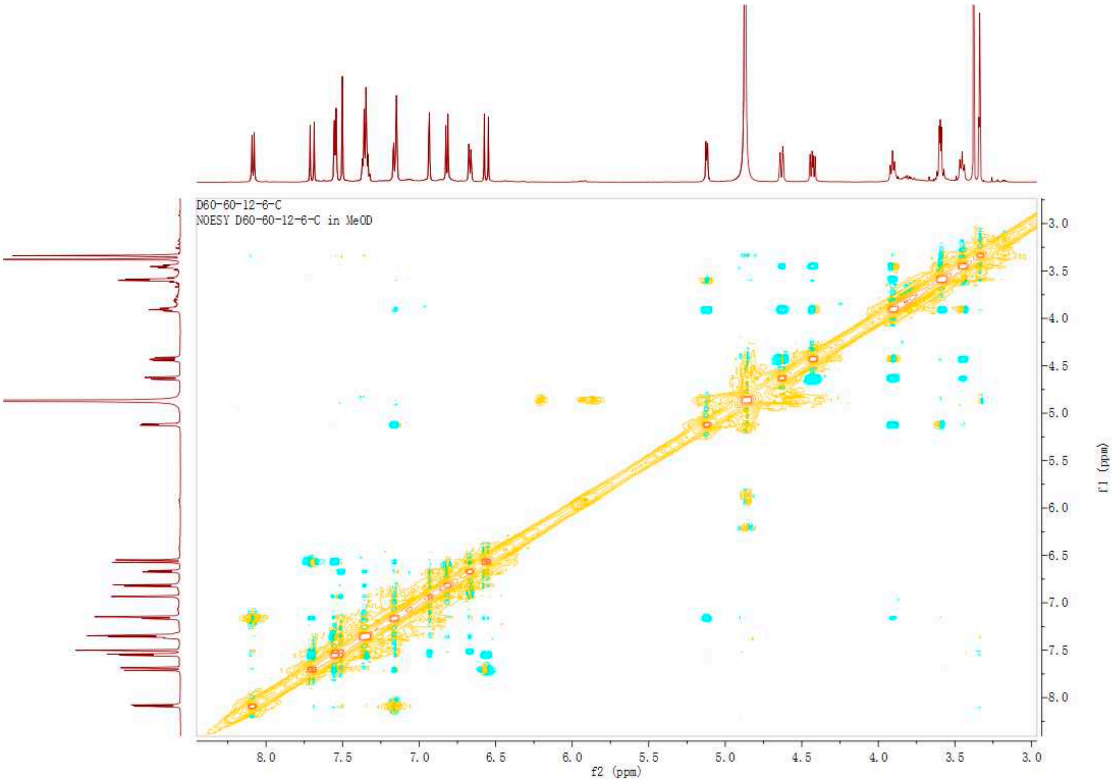

Figure S15. NOESY spectrum of the new compound **2.**

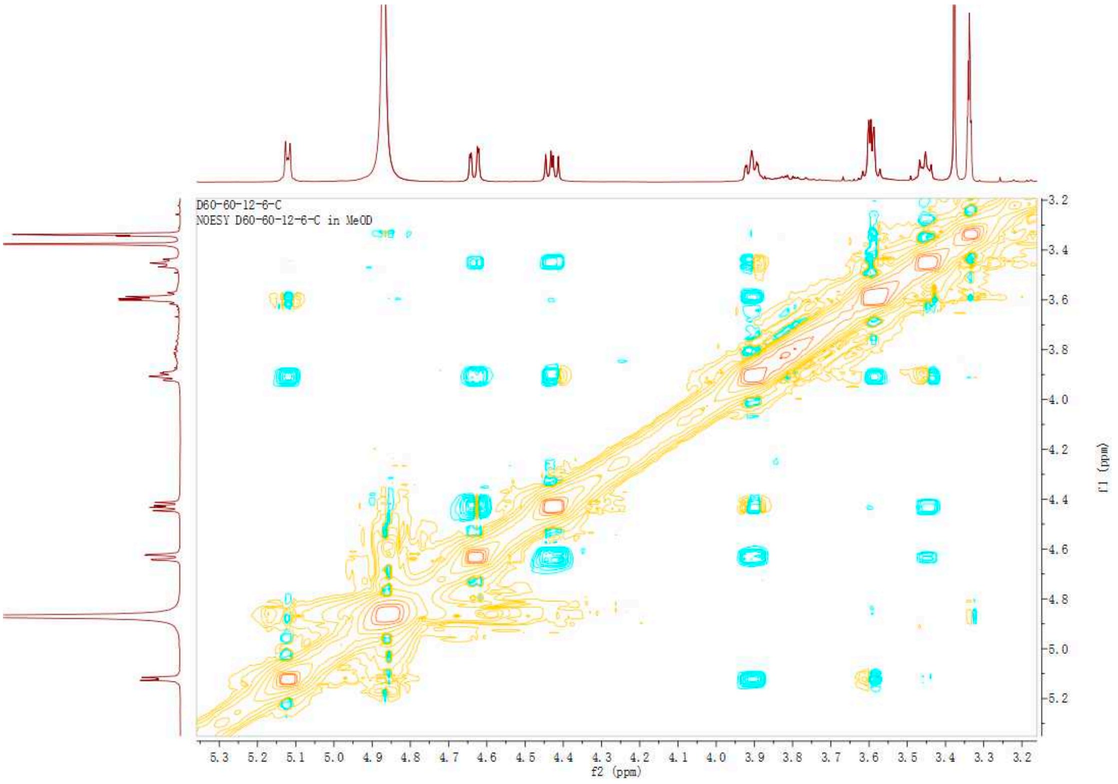

Figure S16. Enlarged NOESY spectrum of the new compound **2.**

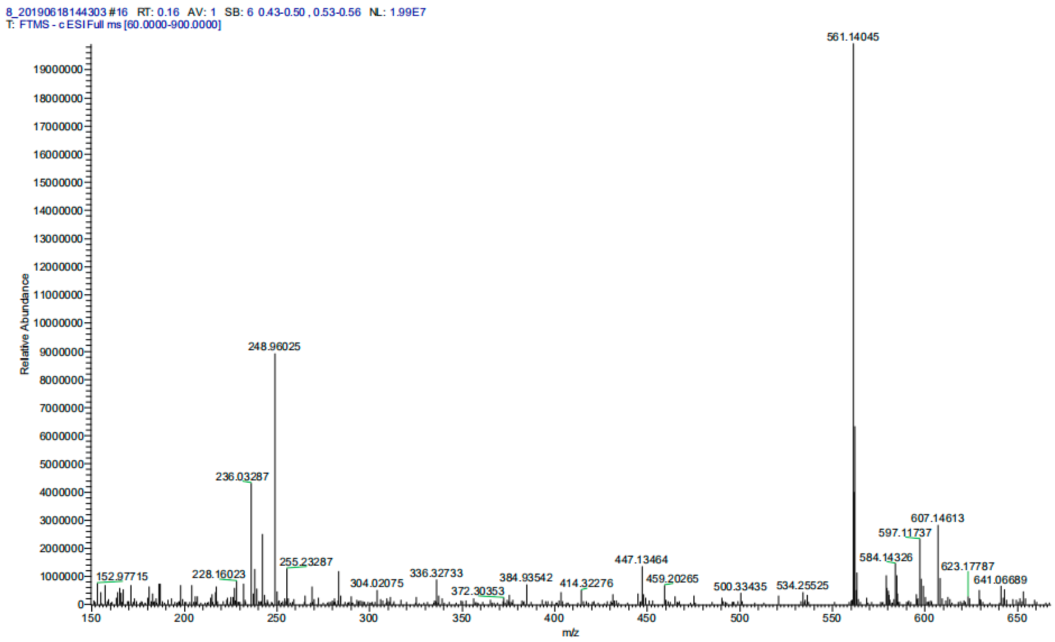

Figure S17. HRESIMS spectrum of the new compound **2.**

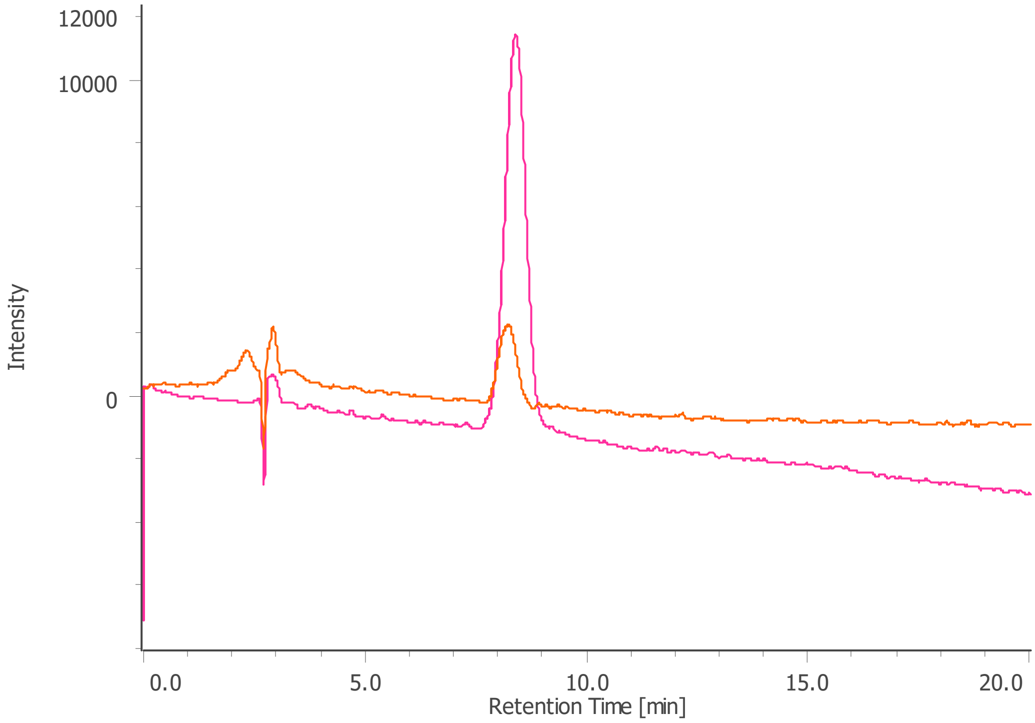

Figure S18. The chromatogram of the glucose standard and hydrolyzation product of **2**

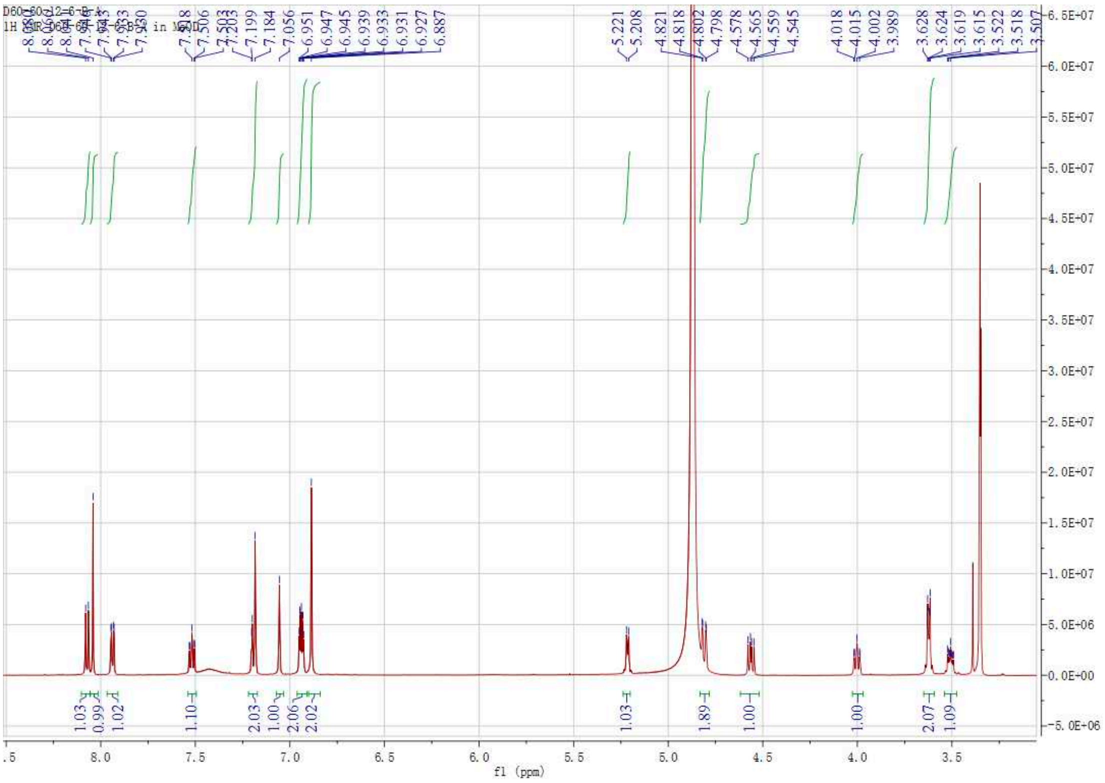

Figure S19. ^{1}H NMR (600 MHz, CD_3OD) spectrum of the new compound **3.**

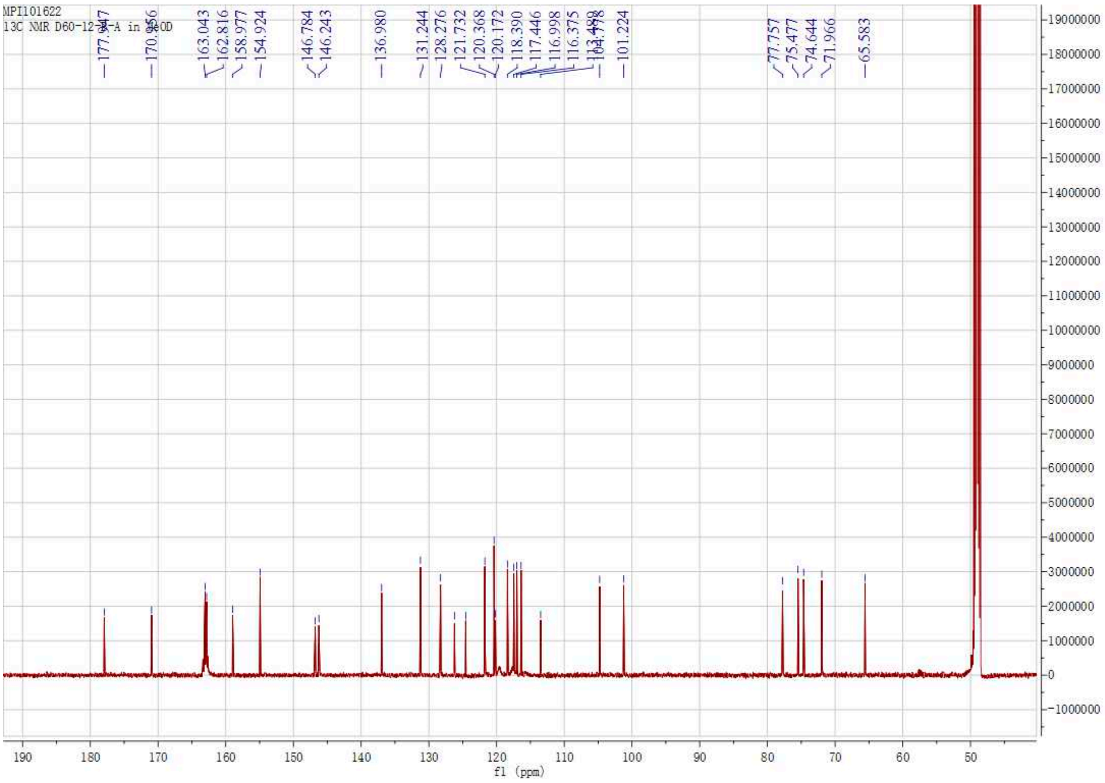

Figure S20. ^{13}C NMR (150 MHz, CD_3OD) spectrum of the new compound **3.**

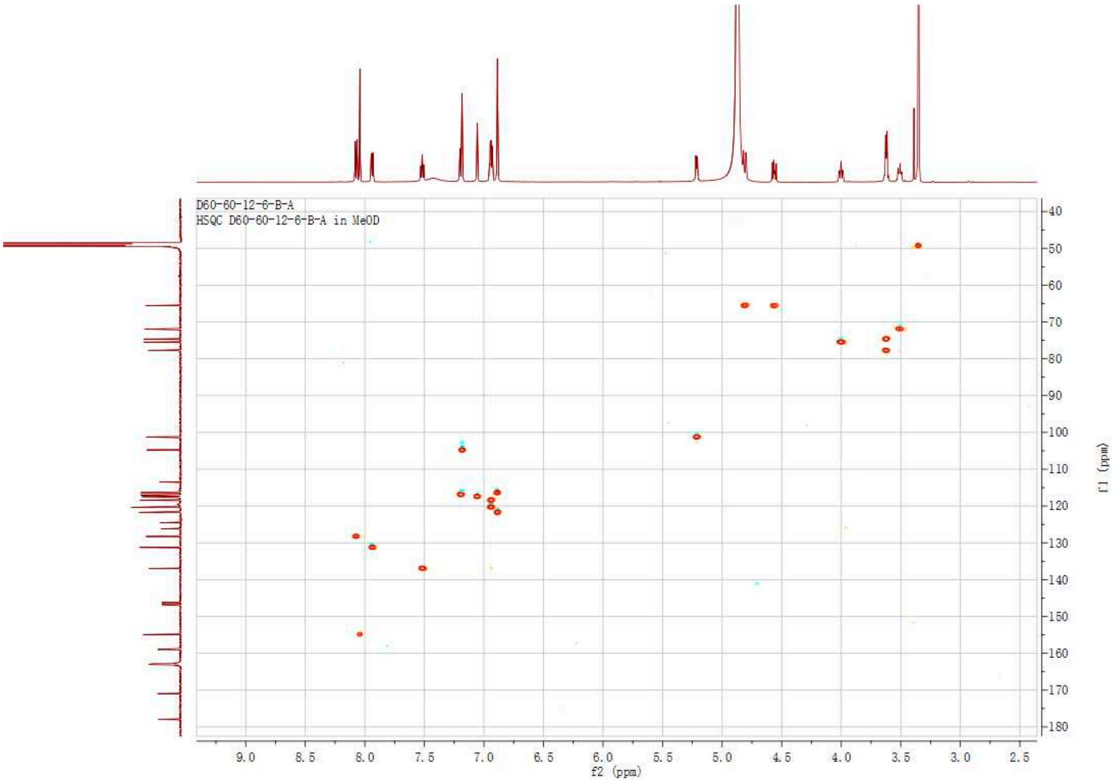

Figure S21. HSQC spectrum of the new compound **3.**

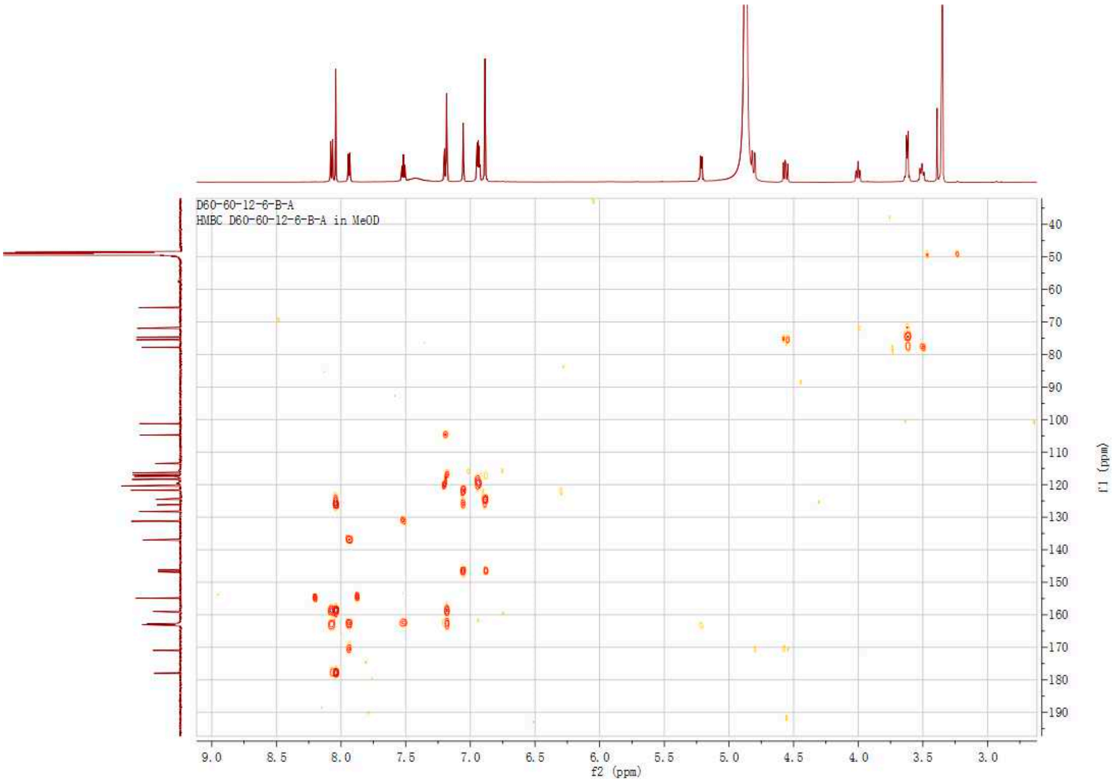

Figure S22. HMBC spectrum of the new compound **3.**

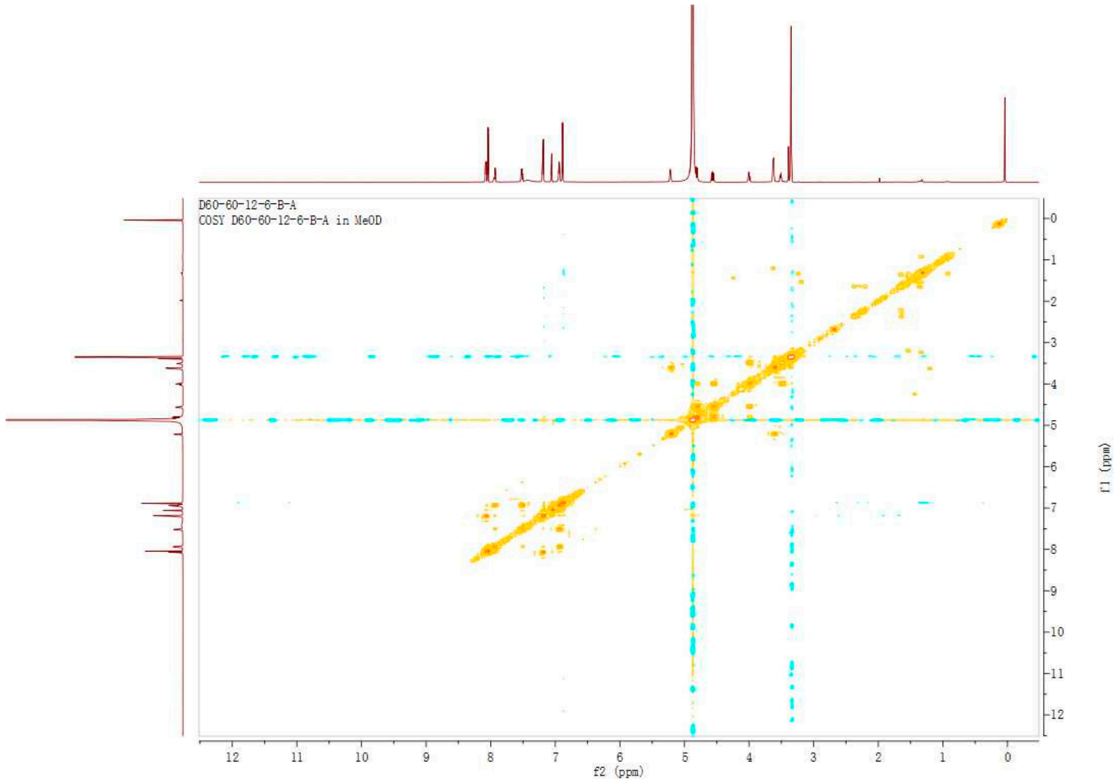

Figure S23. ^{1}H-^{1}H COSY spectrum of the new compound **3.**

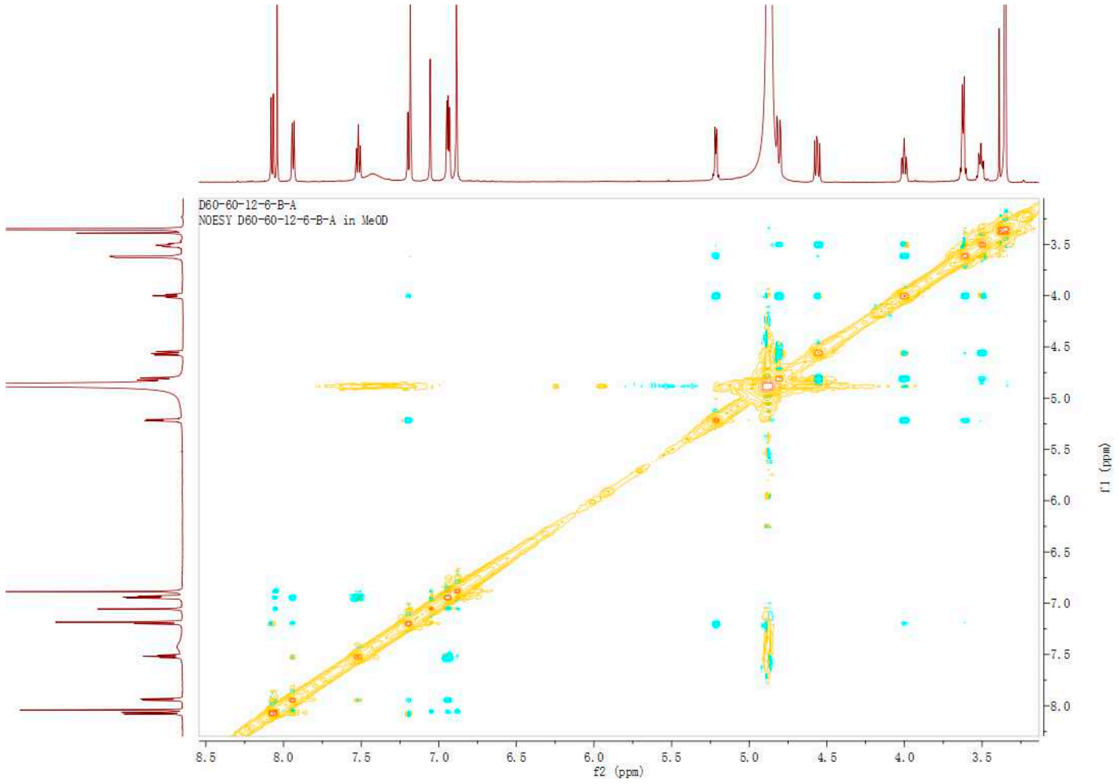

Figure S24. NOESY spectrum of the new compound **3.**

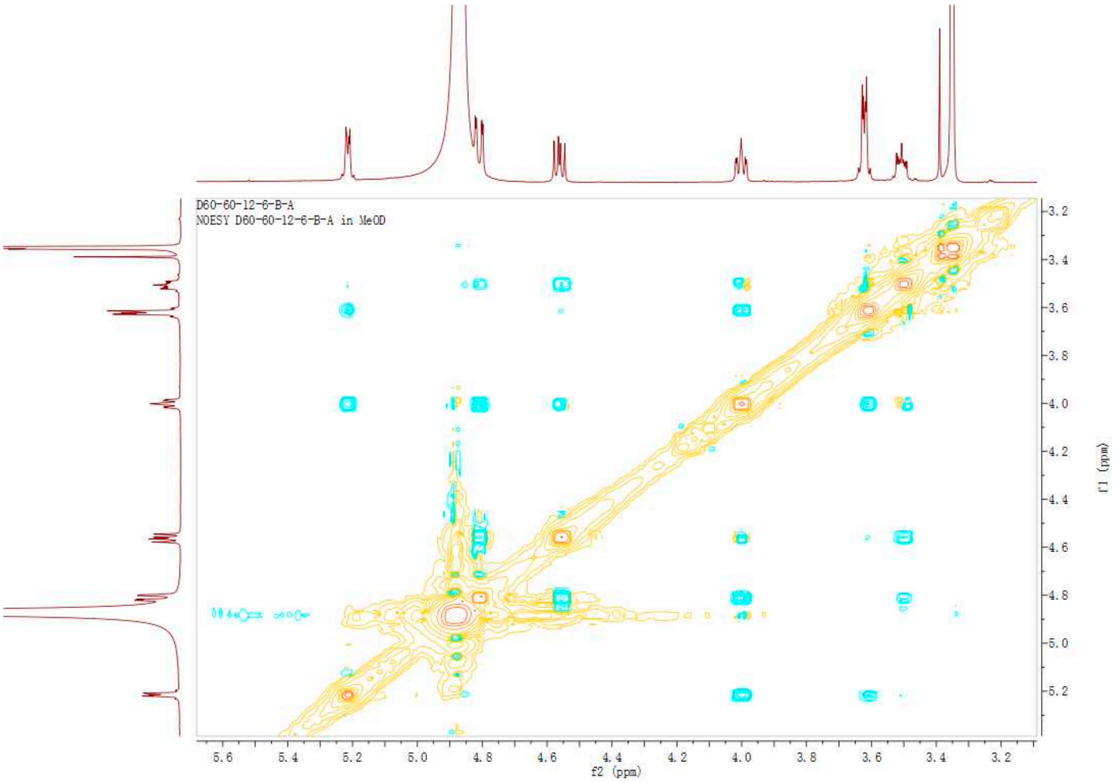

Figure S25. Enlarged NOESY spectrum of the new compound **3.**

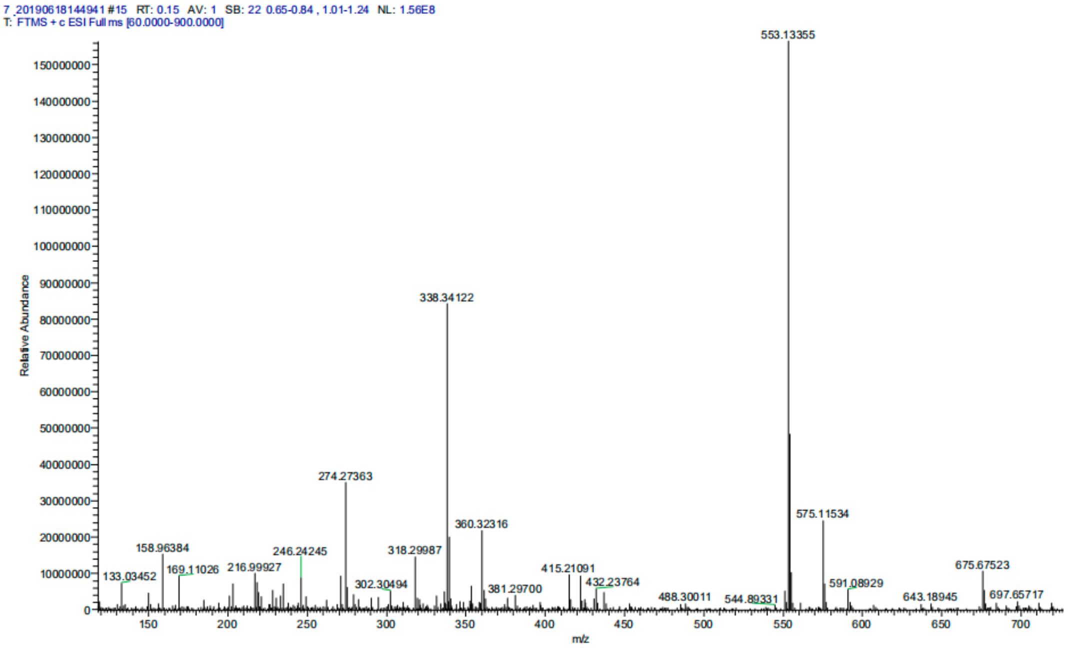

Figure S26. HRESIMS spectrum of the new compound **3.**

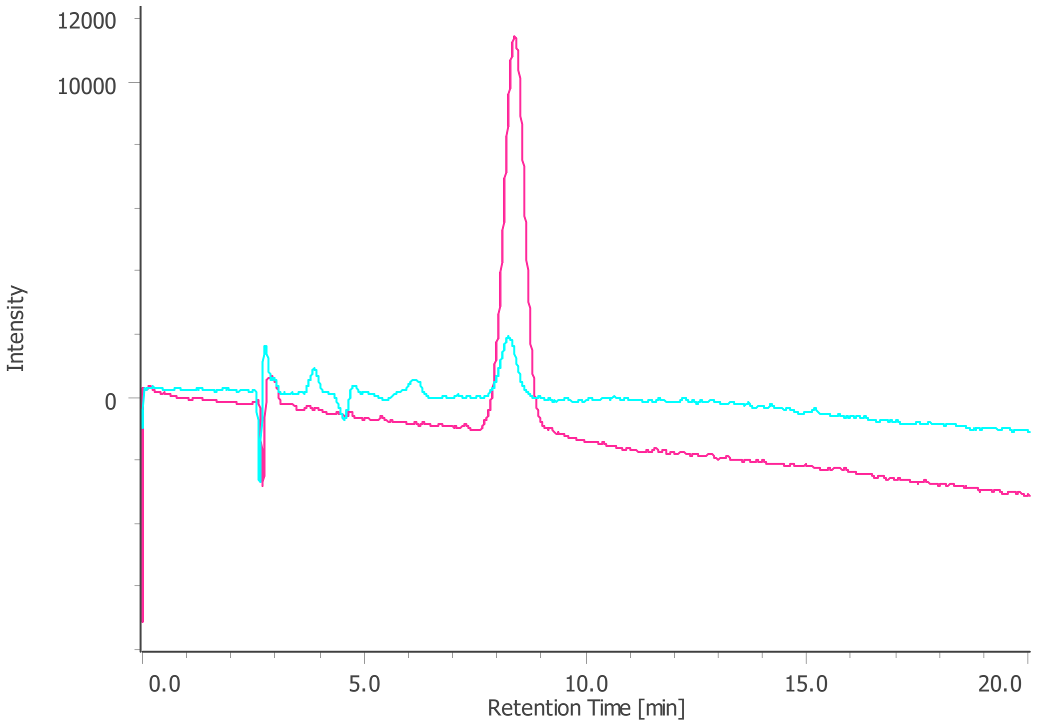

Figure S27. The chromatogram of the glucose standard and hydrolyzation product of **3**

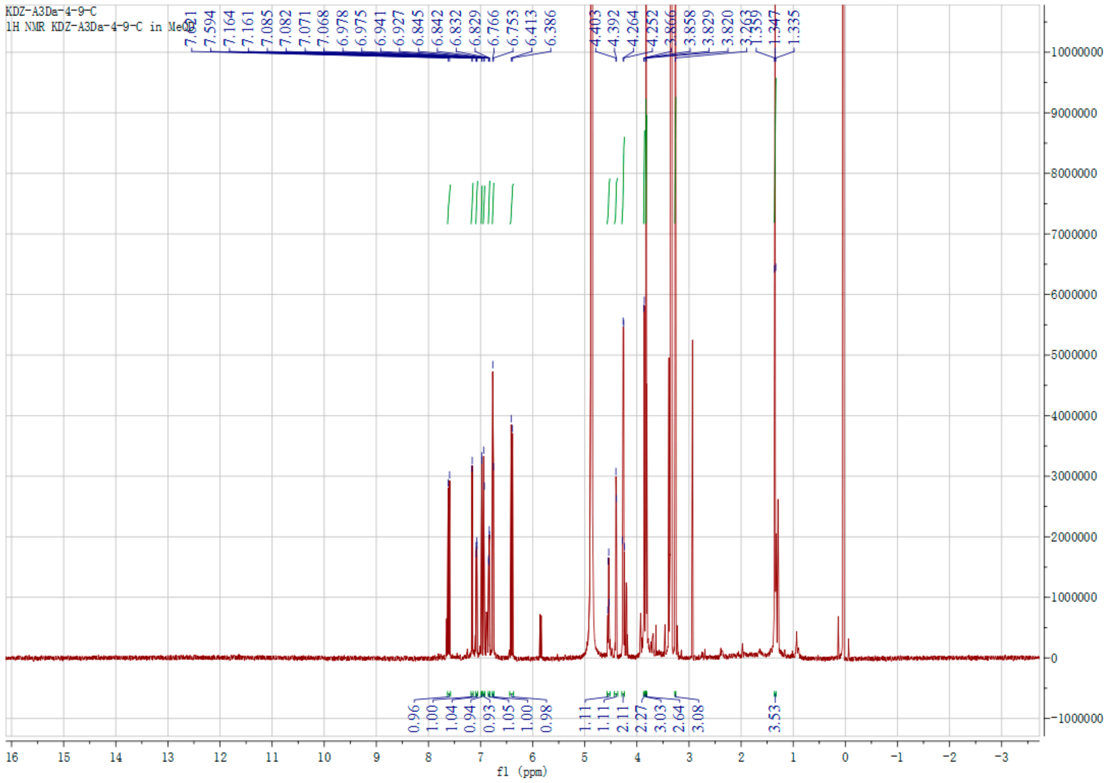

Figure S28. ^{1}H NMR (600 MHz, CD_3OD) spectrum of the new compound **4**.

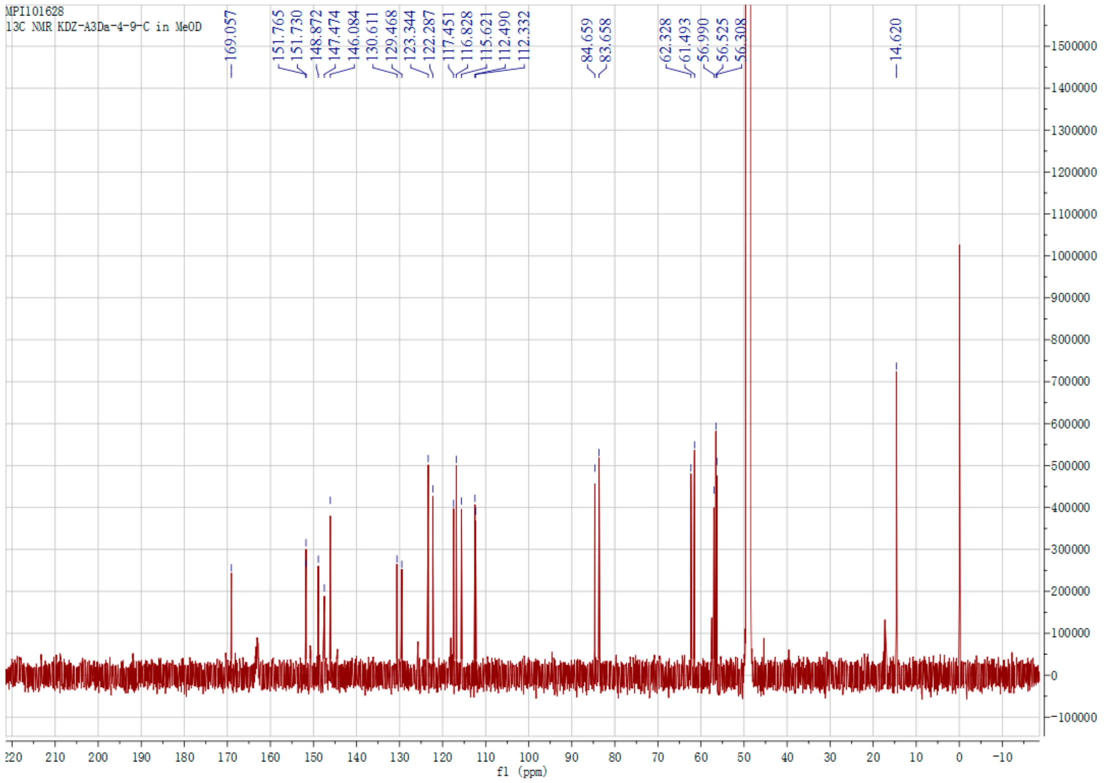

Figure S29. ^{13}C NMR (150 MHz, CD_3OD) spectrum of the new compound **4.**

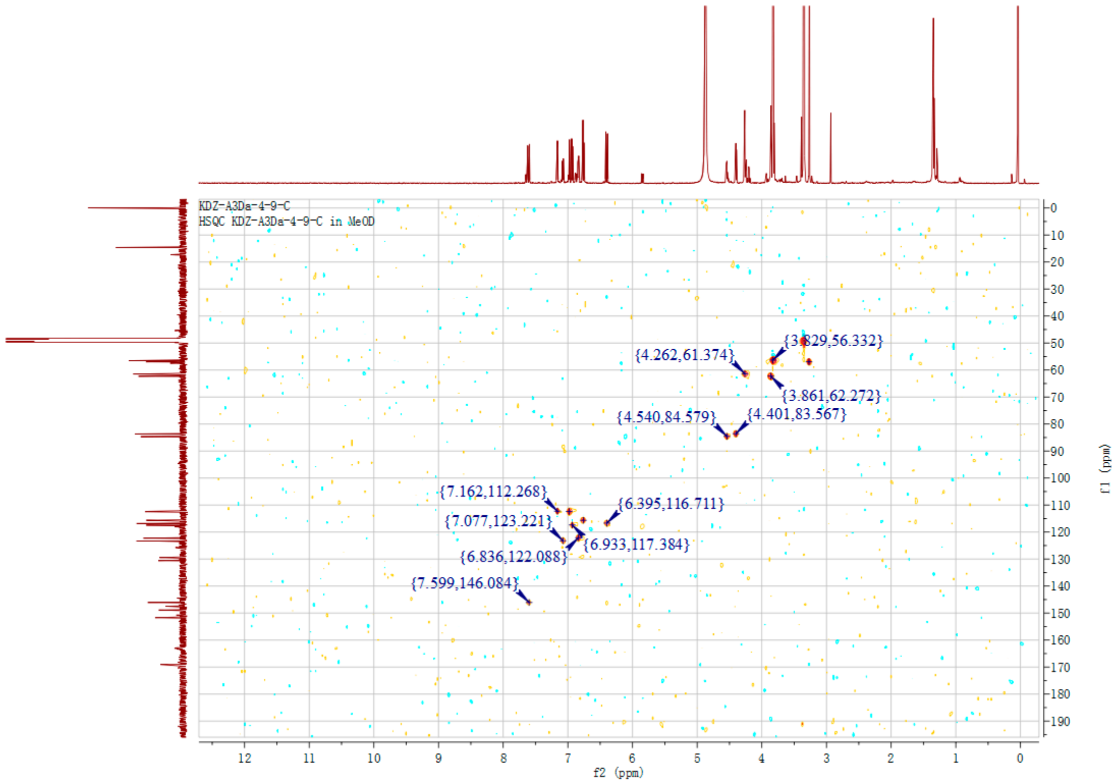

Figure S30. HSQC spectrum of the new compound **4.**

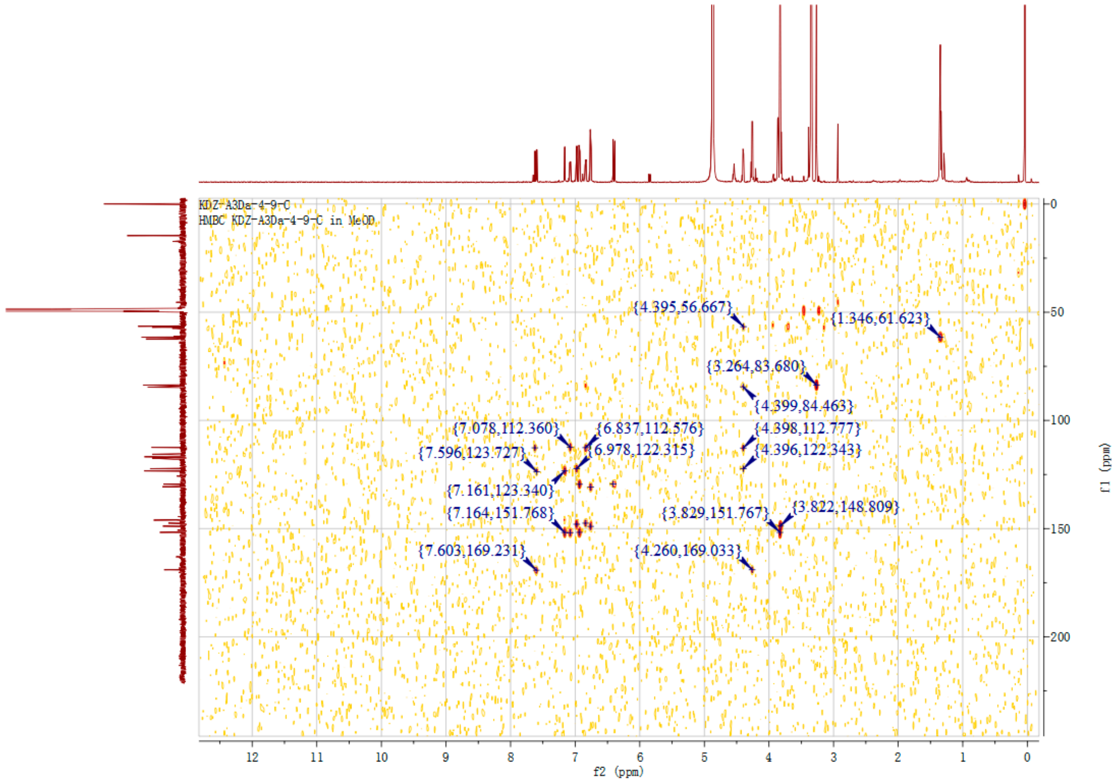

Figure S31. HMBC spectrum of the new compound **4**.

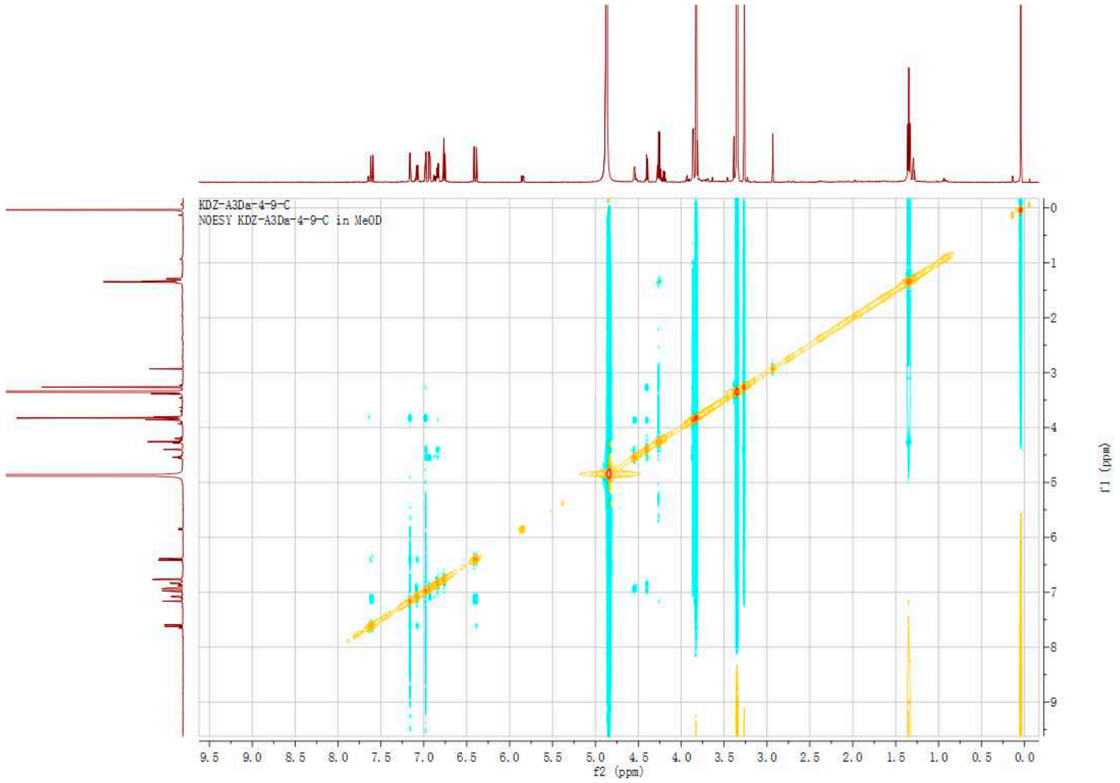

Figure S32. NOESY spectrum of the new compound **4**.

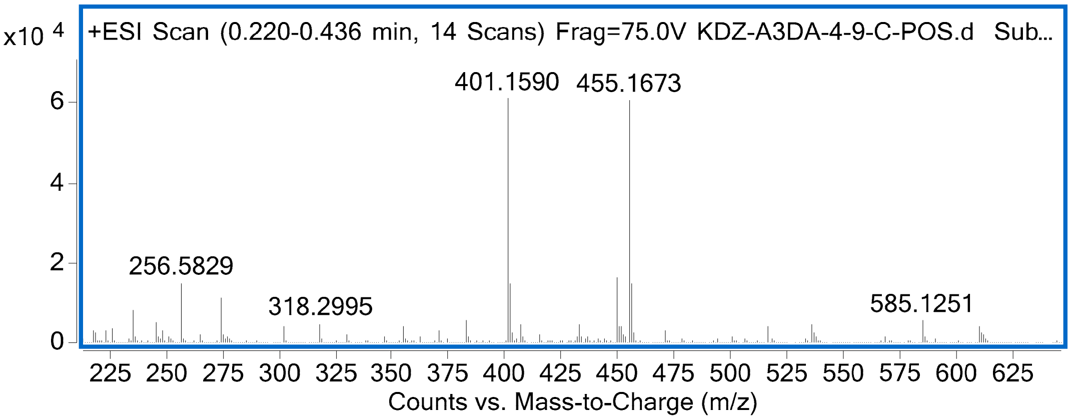

Figure S33. HRESIMS spectrum of the new compound **4.**

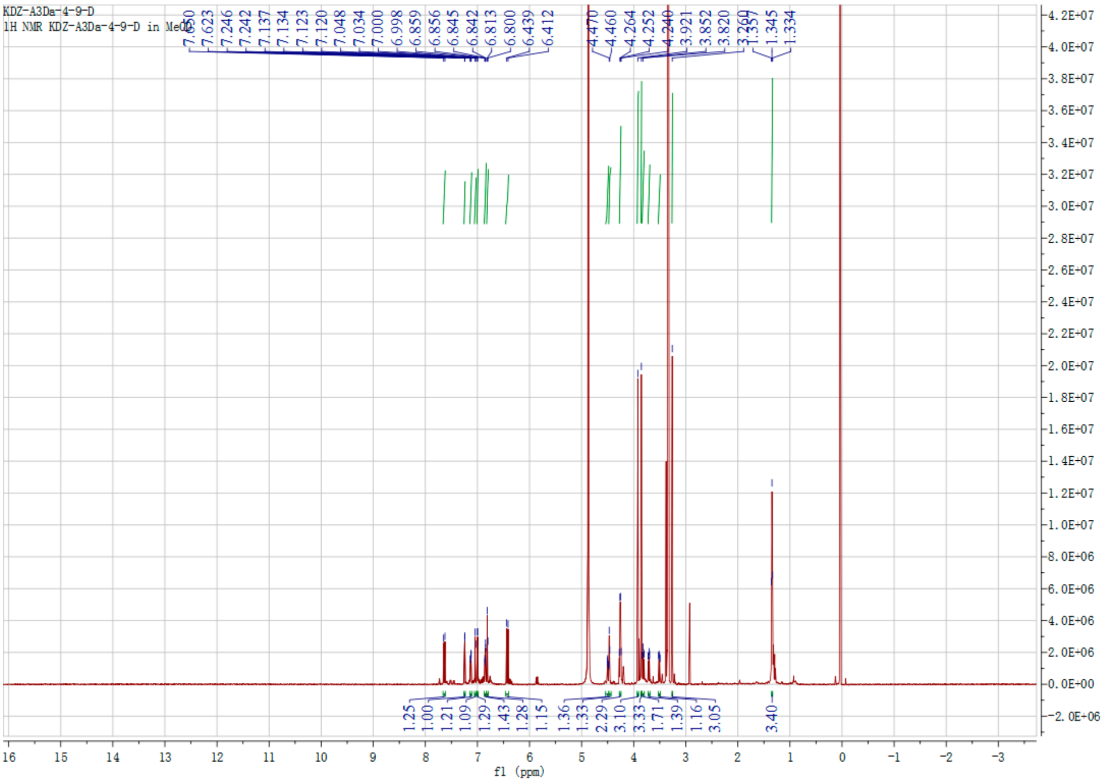

Figure S34. ^{1}H NMR (600 MHz, CD_3OD) spectrum of the new compound **5.**

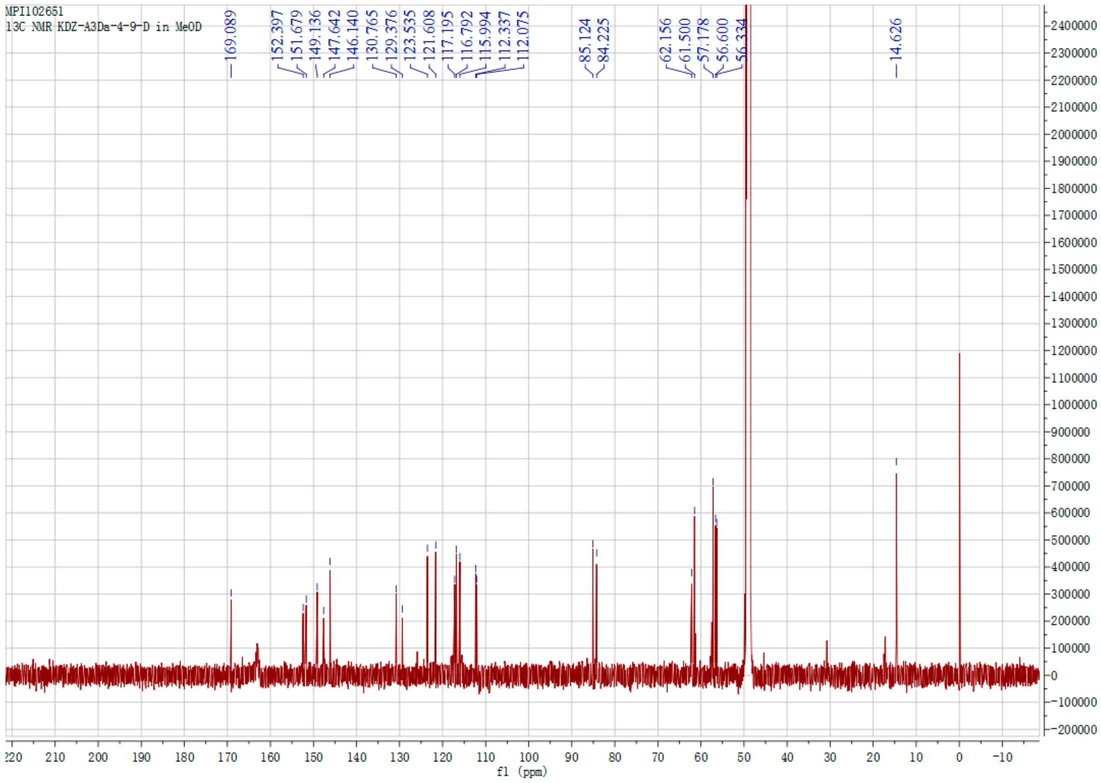

Figure S35. ^{13}C NMR (150 MHz, CD_3OD) spectrum of the new compound **5.**

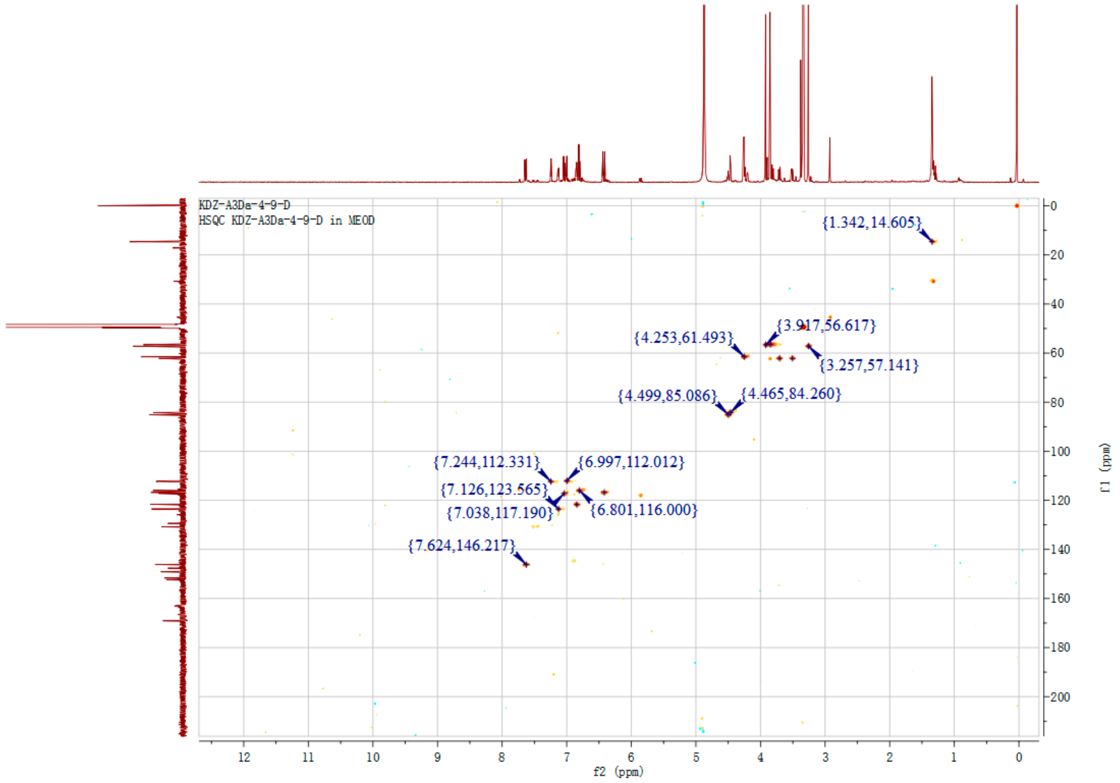

Figure S36. HSQC spectrum of the new compound **5.**

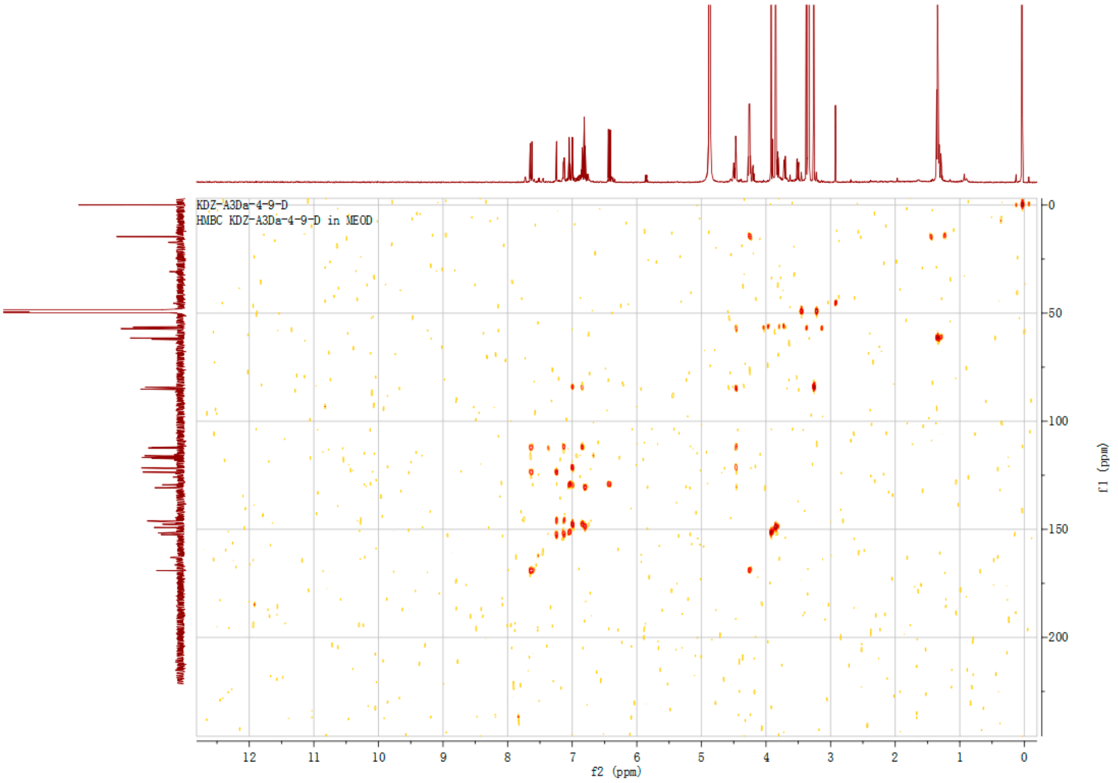

Figure S37. HMBC spectrum of the new compound **5.**

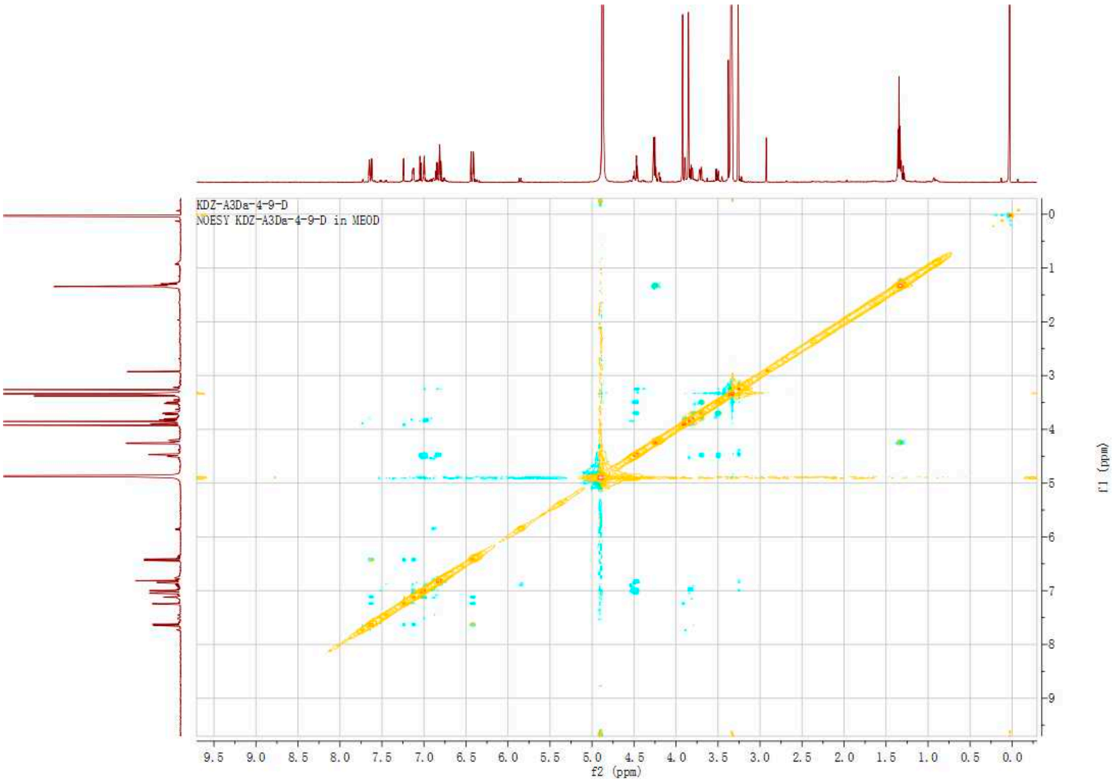

Figure S38. NOESY spectrum of the new compound **5.**

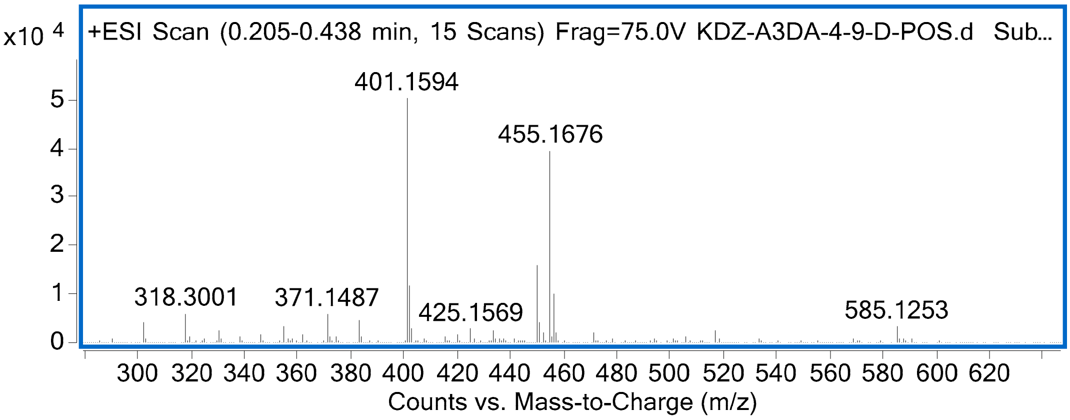

Figure S39. HRESIMS spectrum of the new compound **5.**
